# Are two stresses better than one? In-depth analysis of 5 Arabidopsis accessions under water and nitrogen stress

**DOI:** 10.1093/plcell/koae174

**Published:** 2024-06-25

**Authors:** Shanice S Webster

**Affiliations:** Assistant Features Editor, The Plant Cell, American Society of Plant Biologists; Howard Hughes Medical Institute, Chevy Chase, MD 20815, USA; Duke University Department of Biology, Durham, NC 27708, USA

Plant species are simultaneously affected by a broad range of environmental stresses, such as low soil nutrients and decreased water availability. Although much is known about the effects of individual stresses, we have less understanding of the plant response to simultaneous stresses (Araus et al. 2020), and the effects of such multifactorial stresses cannot be predicted from the individual stresses involved (Zandalinas et al. 2021). Multifactorial stress events can seriously affect plant growth and survival, as well as increase plant susceptibility to pathogens, even when the extent of the individual stresses alone might have little to no effect (Zandalinas et al. 2021). However, new work by **Zeyun Xue and colleagues (Xue et al. 2024)** presents an intriguing alternative to this model, where combined stresses might alleviate the effects of a single stress. The group presents an in-depth transcriptome and metabolome analysis of individual and combined stresses of nitrogen and water deficiency conditions in natural variants of *Arabidopsis thaliana*. Their study provides insights into the fine-tuned responses at the transcriptional and metabolic levels and sheds light on the impact of combined stresses on plant molecular programming.

Xue et al. monitored continuous plant growth for 5 accessions under the single stresses of low nitrogen or drought and the combined stress of low nitrogen and drought, with adequate nitrogen and water used as the control condition. Using a “Phenoscope,” a phenotyping robot that performs high-throughput phenotyping of hundreds of plants, the authors monitored rosette biomass for 31 days. The authors observed that plants grown under the single low nitrogen stress were one-half the size of plants in control conditions, while plants grown under drought stress alone (with adequate nitrogen) were two-thirds the size of control plants. Interestingly, plants simultaneously subjected to both stresses had rosette sizes similar to that of the low-nitrogen-only stress, suggesting that nitrogen deficiency was the dominating factor of the combined stresses (see Figure). Looking at the rosette growth rates over time, the combined stress conditions showed a pattern that combined those of the single stresses. In drought conditions, plant growth transiently decreased at 17 days with subsequent recovery to control levels, whereas in low-nitrogen conditions, plant growth rate gradually decreased with no recovery. In the combined stress conditions, growth rates transiently reduced and then recovered similar to the drought-only condition, followed by a gradual decrease at the later phase similar to the low-nitrogen-only stress (see Figure). Together, these data indicate that on a macroscopic scale, plants respond and integrate individual environmental stresses to eventually tailor a unique output response to the combined stress.

**Figure.**
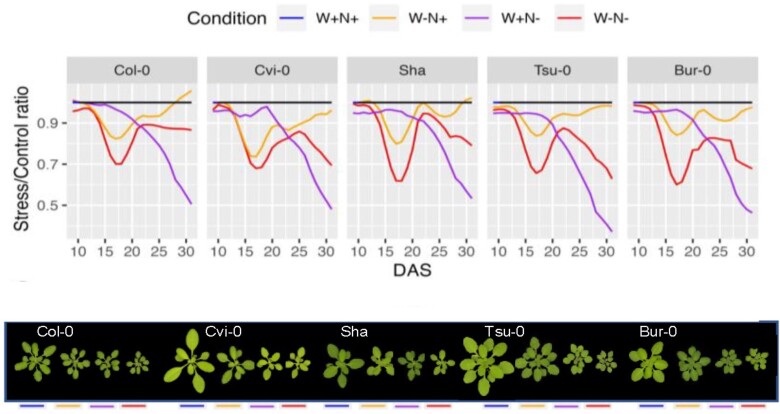
Rosette growth in response to mild drought and nitrogen deficiency. Dynamics of Relative Expansion Rate normalized by control condition (upper panel) and typical rosette appearance for each accession at 31 DAS (lower panel). DAS, days after sowing; PRA, projected rosette area. Adapted from Xue et al. (2024), Figure 1.

To delve into the molecular mechanisms of single and combined stress responses, the authors investigated the transcriptome and metabolome profiles of each plant accession under the different stress treatments. The low nitrogen alone, drought alone, and combined stress treatments were associated with 228, 1376, and 1008 differentially expressed genes (DEGs), respectively. Gene ontology analysis showed that nitrate responses, anthocyanins, and salicylic acid pathways were highly enriched across all 3 treatments. Several known nitrogen deficiency stress response genes were differentially expressed, including several glutaredoxins such as *ROXY11* and *NIGT1.1*, in single nitrogen stress and combined stress. Under the combined stress, auxin and carbohydrate biosynthesis–related transcripts were differentially expressed compared to control conditions. The group's metabolome analysis further differentiated between single and combined stress treatments. Although low nitrogen was found to be the dominating stress on plant growth under combined conditions, these analyses suggested that the transcriptional and metabolome responses due to nitrogen deficiency were attenuated when combined with drought conditions. Intriguingly, all 5 accessions show DEGs with lower fold changes in the combined stress condition compared with low nitrogen alone. Similarly, the magnitude of metabolite accumulation was lower under the combined stress compared with low nitrogen alone.

How might drought lessen the impact of nitrogen stress? One explanation could be the decreased plant biomass observed in the single water stress conditions, leading to reduced photosynthesis and therefore lower nitrogen demand, mitigating the effects of low soil nitrogen (Cramer et al. 2009). However, it is also possible that the smaller plants, due to low nitrogen stress, did not experience the same drought stress effect as the larger control plants, and a more detailed physiological analysis of the drought and low nitrogen response, including, for example, leaf and root water potentials, root growth, and cellular parameters, might be needed to tease apart these effects. Regarding the transcriptome data, all accessions showed an attenuation of the nitrogen deficiency response by drought, but to a variable extent, and stress combination-specific transcripts were not commonly shared among accessions. Overall, it is clear that plant responses to limited water and limited nitrogen are deeply entangled with one another, and natural variation combined with -omics studies can begin to untangle the underlying molecular bases of plant response.
